# SM22*α* Loss Contributes to Apoptosis of Vascular Smooth Muscle Cells via Macrophage-Derived circRasGEF1B

**DOI:** 10.1155/2021/5564884

**Published:** 2021-03-16

**Authors:** Pin Lv, Ya-Juan Yin, Peng Kong, Li Cao, Hao Xi, Ning Wang, Hong-Chao Yang, Yu-Hong Lv, Ning Chen, Rong Wang, Yong-Qing Dou, Hai-Yue Wang, Xiao-Ting Ma, Yan-Ling Lin, Lei Nie, Yan Zhang, Fan Zhang, Mei Han

**Affiliations:** ^1^Department of Biochemistry and Molecular Biology, College of Basic Medicine, Key Laboratory of Medical Biotechnology of Hebei Province, Hebei Medical University, Shijiazhuang, Hebei 050017, China; ^2^The First Hospital of Hebei Medical University, Shijiazhuang, Hebei 050031, China; ^3^Hebei Food Inspection and Research Institute, Hebei Food Safety Key Laboratory, Shijiazhuang, Hebei Province 050227, China

## Abstract

Vascular smooth muscle cell (VSMC) apoptosis is a major defining feature of abdominal aortic aneurysm (AAA) and mainly caused by inflammatory cell infiltration. Smooth muscle (SM) 22*α* prevents AAA formation through suppressing NF-*κ*B activation. However, the role of SM22*α* in VSMC apoptosis is controversial. Here, we identified that SM22*α* loss contributed to apoptosis of VSMCs via activation of macrophages. Firstly, deficiency of SM22*α* enhanced the interaction of VSMCs with macrophages. Macrophages were retained and activated by *Sm22α*^−/−^ VSMCs via upregulating VCAM-1 expression. The ratio of apoptosis was increased by 1.62-fold in VSMCs treated with the conditional media (CM) from activated RAW264.7 cells, compared to that of the control CM (*P* < 0.01), and apoptosis of *Sm22α*^−/−^ VSMCs was higher than that of WT VSMCs (*P* < 0.001). Next, circRasGEF1B from activated macrophages was delivered into VSMCs promoting ZFP36 expression via stabilization of ZFP36 mRNA. Importantly, circRasGEF1B, as a scaffold, guided ZFP36 to preferentially bind to and decay Bcl-2 mRNA in a sequence-specific manner and triggered apoptosis of VSMCs, especially in *Sm22α*^−/−^ VSMCs. These findings reveal a novel mechanism by which the circRasGEF1B-ZFP36 axis mediates macrophage-induced VSMC apoptosis via decay of Bcl-2 mRNA, whereas *Sm22α*^−/−^ VSMCs have a higher sensitivity to apoptosis.

## 1. Introduction

Vascular smooth muscle cells (VSMCs) are the main structural cells of blood vessels, and damage or death of VSMCs contributes to multiple vascular pathologies. VSMC phenotypic switching after injury is extensive and has been ascribed to the injury stimulus; in many cases, VSMC phenotypic switching is accompanied by changes in cell proliferation, cell migration, inflammation, and apoptosis. In atherosclerosis, apoptosis of VSMCs has been associated with plaque rupture and aneurysm formation, which are thought to be a result of chronic inflammation [1]. Furthermore, VSMC apoptosis is implicated in medial degeneration seen in a variety of human genetic diseases including Marfan's syndrome. Both inflammatory cell infiltration and proinflammatory cytokine stimulation induce VSMC apoptosis [2, 3], suggesting that VSMCs are more prone to apoptosis in inflammatory microenvironment.

A decrease in VSMC marker genes, including smooth muscle (SM) 22*α* and SM *α*-actin, that is a prominent feature of VSMC phenotypic switching, has been demonstrated in an advanced human abdominal aortic aneurysm (AAA) and mouse model [4, 5]. Our previous studies show that the arteries of *Sm22α*^−/−^ mice develop enhanced inflammatory response and ROS production, which was involved in neointimal hyperplasia through different signaling mechanisms [6–9], suggesting that *Sm22α*^−/−^ VSMCs have transited to an inflammatory phenotype; however, it is not clear if the modulated VSMCs signal to induce apoptosis. A more recent study using AAV carrying SM22*α* siRNA or SM22*α* overexpression plasmid in Ang II-perfused *ApoE^−/−^* mice confirmed that the causative role of SM22*α* deficiency in AAA formation occurs partly through enhancing vascular inflammation rather than increasing cell apoptosis [10]. However, these findings based on *ApoE*^−/−^ mice with AAV-mediated knockdown or overexpression of SM22*α in vivo* are not enough to exclude a potential causative link between disturbed SM22*α* expression and VSMC apoptosis.

In the present study, we demonstrate that VSMCs of *Sm22α*^−/−^ mice signaled to macrophages and displayed higher sensitivity to apoptosis induced by macrophages. Macrophage-derived circRasGEF1B reprograms VSMCs to apoptosis via directing ZFP36 to selectively bind to and decay Bcl-2 mRNA *in vitro* and *in vivo*. Our findings suggest that inflammatory VSMCs favor interacting with macrophages and the resulting activation of the circRasGEF1B-ZFP36 axis is a novel mechanism underlying macrophages inducing VSMC apoptosis.

## 2. Materials and Methods

### 2.1. Experimental Animals

The *Sm22α^−/−^* mouse line (B6.129S6-*Tagln*^tm2(cre)Yec^/J) carrying a Cre-recombinase gene inserted into the endogenous SM22*α* locus was purchased from the Jackson Laboratory. All animal procedures conformed to the *Guide for the Care and Use of Laboratory Animals* published by the US National Institutes of Health and were approved by the Institutional Animal Care and Use Committee of Hebei Medical University.

### 2.2. Complete Secretome Analysis

Total serum-free media obtained from WT and *Sm22α^−/−^* VSMCs were collected at 4°C, and protease inhibitor cocktail tablet (Roche Inc.) was added to total conditioned media to prevent protein degradation. The protein concentration of each sample was determined using the Bradford assay (Bio-Rad). 200 *μ*g of protein sample was used for mass spectrometry analysis. A filter-aided sample preparation (FASP) protein digestion protocol was used for sample preparation [11], and reactions were carried out on a 10 kDa MWCO filter (UFC500396, Amicon Ultra). Protein alkylation, digestion, iTRAQ labeling, offline 2D LC-MS/MS analysis, proteomic data, and bioinformatics analysis were performed as described previously [12]. To screen adhesion-related differential proteins in VSMC media between WT and *Sm22α^−/−^*, significantly enriched proteins were identified by Gene Ontology (GO) analysis.

### 2.3. RNA-seq Data Analysis

Raw sequencing reads are available from the Gene Expression Omnibus under GEO accession number GSE99811, and data analysis was performed as described previously [13]. To screen apoptosis-related differential genes, we identified significantly enriched GO functional categories in the sets of genes up- and downregulated relative to control in circRasGEF1B-knockdown cells.

### 2.4. Ang II-Induced Abdominal Aortic Aneurysm (AAA) Model

Ten- to 12-week-old male *Sm22α^−/−^* and WT mice were used for the experiments. Osmotic minipumps (ALZET, model 2004) were filled with saline or angiotensin (Ang) II (A9525, Sigma-Aldrich) at a dosage of 1000 ng/kg/min dissolved in saline. The pumps were placed into the subcutaneous space for 4 weeks. After the mice were sacrificed, the aorta was dissected free from the surrounding connective tissue. Pictures were taken with a digital camera and used to measure the outer diameter of the suprarenal aorta as described previously [14]. Suprarenal regions of the abdominal aorta were identified between the last pair of intercostal arteries and the right renal branch. The maximum width of the abdominal aorta was analyzed using Image-Pro Plus software after adjusting the scale according to the ruler in aorta pictures. A mean of three measurements was used. AAA in mice was defined as a 50% or greater increase in the external width of the suprarenal aorta compared with aortas from the controls [15].

### 2.5. Histological Analyses

For immunohistochemical staining, frozen sections were incubated with 3% hydrogen peroxide, followed by blocking with 3% normal blocking serum. The sections were incubated with primary antibodies against CD14 (1 : 500 dilution, ab182032, Abcam) or SM *α*-actin (1 : 500 dilution, ab124964, Abcam) at 4°C overnight, followed by a secondary antibody before staining with the DAB Kit (ZSGB-BIO, Beijing, China). Nuclei were counterstained with hematoxylin. Sections incubated with species-matched IgG alone were used as negative controls.

For immunofluorescence staining, frozen aortic sections were incubated with antibodies against CD68 (1 : 500 dilution, ab955, Abcam), followed by TRITC-conjugated secondary antibodies. The fluorescence signal was monitored by confocal laser scanning microscopy (Leica SP5, Switzerland).

### 2.6. Cell Culture and Treatment

Primary VSMCs from the aortas of WT or *Sm22α^−/−^* mice (8-12 weeks old, male) were isolated by collagenase digestion. The isolated cells were maintained in low-glucose Dulbecco's modified Eagle's medium (DMEM; Invitrogen) containing 10% fetal bovine serum (FBS; Gibco), 100 U/mL penicillin, and 100 *μ*g/mL streptomycin [16, 17]. The purity of VSMCs was verified by immunofluorescence staining of SM *α*-actin. Cells from passages 3 to 5 only were used for further experiments. The mouse VSMC line MOVAS was purchased from ATCC (CRL-2797) and cultured in high-glucose DMEM containing 10% FBS and 0.2 mg/mL G-418. Before being treated with different stimulations, all of the VSMCs were incubated in serum-free medium for 24 h. RAW264.7 cells were purchased from ATCC (TIB-71™) and cultured in low-glucose DMEM containing 10% FBS, 100 U/mL penicillin, and 100 *μ*g/mL streptomycin.

### 2.7. Adenovirus Packaging and Infection

Full-length cDNA of SM22*α* was cloned into pCMV-FLAG®-MAT-Tag™-1 Expression Vector (C5864; Sigma-Aldrich). The pAdeno-MCMV-HA-P2A-EGFP was used to pack green fluorescent protein- (GFP-) tagged adenovirus (pAdeno-MCMV-Flag-SM22*α*-Mat-P2A-EGFP, Ad-SM22*α* for short). The VSMCs were infected with 10^10^ pfu/mL adenovirus for 24 h, washed, maintained in serum-starved medium for 24 h, and then treated with indicated stimulations.

### 2.8. Small Interfering RNA (siRNA) Transfection

The siRNA duplexes targeting mouse circRasGEF1B (si-circRasGEF1B), ZFP36 (si-ZFP36), and scrambled siRNA (si-Con) were designed and obtained from RiboBio (Guangzhou, China); the siRNAs were transiently transfected into VSMCs using Lipofectamine® RNAiMAX Transfection Reagent (Invitrogen) according to the manufacturer's protocol.

### 2.9. Plasmid Transfection

The circRasGEF1B sequence was amplified by PCR and constructed into a pLCDH-ciR vector (Geenseed Biotech, Guangzhou, China). The different sequences of circRasGEF1B deletion mutants were synthesized and inserted into the pLCDH-ciR vector to overexpress mutant circRasGEF1B. The pLCDH empty vector and pLCDH-circRasGEF1B or pLCDH-circRasGEF1B mutants were transfected into VSMCs with the X-tremeGENE HP DNA transfection reagent (06366236001, Roche) for 24 h according to the manufacturer's protocol and then treated with indicated stimulations.

### 2.10. Migration Assay

Macrophage migratory activity was performed using 24-well transwell plates with a 5 *μ*m pore filter (Corning). VSMCs were pre-seeded in the lower chamber. After achieving confluence, serum-starved VSMCs were stimulated by Ang II (10^−5^ M) for 24 h. Thereafter, RAW264.7 cells were placed in the upper chamber. After incubating for 6 h, nonmigratory cells on the upper membrane surface were removed, and the cells that traversed and spread on the lower membrane surface were fixed with 4% paraformaldehyde and stained with gentian violet. By utilizing a microscope with a 40x objective, the number of migratory cells per membrane was enumerated. At least three random fields in each filter were examined. Each experiment was performed in triplicate, and migration was expressed as the mean ± SD of total cells counted per field.

### 2.11. Adhesion Assay

VSMCs plated in 96-well culture plates were stimulated with Ang II (10^−5^ M) or indicated treatment, and then, RAW264.7 cells (labeled with calcein-AM, Life Technologies) were added to each well. After 30 min incubation, nonadherent cells were removed carefully by washing with cold phosphate-buffered saline (PBS). The fluorescent intensities were determined by excitation and emission at 490 and 535 nm, respectively. For adenovirus-infected VSMCs, RAW264.7 cells were added to each well, and the adherent cells were counted.

### 2.12. Apoptosis Assay

Apoptosis of frozen aortic sections was determined by using the ApopTag Peroxidase *In Situ* Apoptosis Detection Kit (S7100, Chemicon) according to the manufacturer's instructions.

Apoptosis of VSMCs was determined by the Annexin V-FITC/PI Apoptosis Detection Kit (556547, BD Pharmingen) according to the manufacturer's instructions, and the BD LSRFortessa™ flow cytometer (BD Biosciences) was used to analyze the apoptotic index. Alternatively, using the ApopTag Red *In Situ* Apoptosis Detection Kit (Millipore), VSMC apoptosis transfected with plasmids was determined by terminal deoxynucleotidyl transferase-mediated dUTP nick end labeling (TUNEL) staining, and the fluorescence signal was monitored by confocal laser scanning microscopy (Leica SP5, Switzerland).

### 2.13. RNA Isolation and Quantitative Real-Time PCR (qRT-PCR)

Total RNAs were extracted using TRIzol Reagent (Life Technologies), following the manufacturer's instructions. To quantify the amount of mRNA or circRNA, cDNAs were synthesized using the M-MLV First Strand Kit (Life Technologies), and quantitative PCR was performed using SYBR Green qPCR SuperMix-UDG (Life Technologies). For microRNA, total RNA was extracted by using the QIAzol Lysis Reagent. Reverse transcription and quantitative reverse transcription PCR were performed with the miRNA Detection Kit by Sangon Biotech (Shanghai, China). Relative circRNA, mRNA, or miRNA expression was normalized to *β*-actin/GAPDH or U6 snRNA levels, using the 2^−ΔΔCt^ method, respectively. The sequence for each primer is listed in Supplementary Table 1 in Supplementary Materials. The average threshold cycle for each gene was determined from at least three independent experiments.

### 2.14. Western Blot Analysis

Lysates from cells or tissues were prepared with RIPA lysis. Equal amounts of protein were separated by sodium dodecyl sulfate-polyacrylamide gel electrophoresis (SDS-PAGE) and electrotransferred to a polyvinylidene fluoride (PVDF) membrane. Membranes were blocked with 5% nonfat dairy milk and incubated with primary antibodies against anti-VCAM-1 (1 : 1000 dilution, ab134047, Abcam), anti-ZFP36 (1 : 500 dilution, sc-374305, Santa Cruz), anti-Bcl-2 (1 : 1000 dilution, sc-7382, Santa Cruz), anti-Bax (1 : 1000 dilution, sc-7480, Santa Cruz), anti-cleaved caspase-3 (1 : 1000 dilution, ab49822, Abcam), anti-pro-caspase-3 (1 : 1000 dilution, ab32499, Abcam), anti-SM22*α* (1 : 1000 dilution, ab14106, Abcam), anti-ICAM-1 (1 : 1000 dilution, ab222736, Abcam), anti-IGFBP7 (1 : 500 dilution, DF7131, Affinity Biosciences), and anti-TNC (1 : 500 dilution, DF8051, Affinity Biosciences) at 4°C overnight. GAPDH (1 : 1000 dilution, ab181602, Abcam) was used as an internal control. This was followed by incubation with an IRDye800®-conjugated secondary antibody (1 : 20000 dilution, Rockland) for 1 h at room temperature and subsequent scanning with the Odyssey Infrared Imaging System (LI-COR Biosciences). The integrated intensity for each detected band was determined using Odyssey Imager software. Data are presented as mean ± SD from at least three independent experiments.

### 2.15. RNA Immunoprecipitation (RIP)

VSMCs were washed in ice-cold PBS, lysed in lysis buffer (20 mM/L Tris-HCl, pH 7.0, 150 mM/L NaCl, 0.5% NP-40, 5 mM/L EDTA, with freshly added 1 mM/L DTT, 1 mM/L PMSF, and 0.4 U/*μ*L RNase inhibitor), and then incubated with 5 *μ*g ZPF36 primary antibody (ABE285, Merck) at 4°C for 2 h. 50 *μ*L Protein A/G PLUS-Agarose (Santa Cruz) was added to each sample, and the mixtures were incubated at 4°C for 4 h. The pellets were washed with PBS and resuspended in 1 mL TRizol Reagent (Invitrogen). The precipitated RNAs in the aqueous solution were subjected to qRT-PCR analysis to demonstrate the presence of the binding products using respective primers. The experiment was replicated at least three times.

### 2.16. RNA Pull-Down Assay

Eight to ten dishes of 15 cm in diameter of VSMCs were used per RNA pull-down experiment. RNA pull-down assays were performed as described [18]. Briefly, VSMCs were washed in ice-cold PBS with 0.4 U/*μ*L RNase inhibitor, lysed in 500 *μ*L lysis buffer (20 mM/L Tris-HCl, pH 7.0, 150 mM/L NaCl, 0.5% NP-40, 5 mM/L EDTA, with freshly added 1 mM/L DTT, 1 mM/L PMSF, and 0.4 U/*μ*L RNase inhibitor), and then incubated with 3 *μ*g biotinylated DNA oligo probes (designed and obtained from RiboBio, Guangzhou, China) at 4°C for 2 h. A total of 50 *μ*L Dynabeads™ MyOne™ Streptavidin C1 magnetic beads (Invitrogen) were added to each binding reaction and further incubated at 4°C for 4 h. The beads were washed briefly with lysis buffer for three times; then, the enriched proteins were identified by immunoblotting, and enriched RNAs were identified by qRT-PCR. The experiment was replicated at least three times.

### 2.17. Fluorescence In Situ Hybridization (FISH)

The VSMCs were washed in PBS, fixed in 4% paraformaldehyde for 30 min, and permeabilized for 15 min. For FISH, the cells were incubated using specific probes of circRasGEF1B according to user manual of the Fluorescent *In Situ* Hybridization Kit (RiboBio, Guangzhou, China). Hybridization was performed using fluorescence-labeled probes in hybridization buffer by incubation at 37°C for overnight. After stringent washing with SSC buffer, cell nuclei were counterstained with DAPI (Invitrogen). Images were acquired using confocal laser scanning microscopy (Leica SP5, Switzerland).

### 2.18. mRNA Stability Assay

VSMCs overexpressed circRasGEF1B following transfecting with the plasmid for 24 h. Then, de novo RNA synthesis was blocked with 10 *μ*g/mL ActD (C7698, Sigma-Aldrich). Total RNA was harvested at indicated time points, and mRNA expression was detected by qRT-PCR. The half-life of ZFP36 mRNA was determined by comparing to the mRNA level before adding ActD.

### 2.19. Statistical Analysis

Data analysis was performed using SPSS version 16.0 or GraphPad Prism 6 software. Data are presented as the means ± SD from at least three independent experiments, and each independent experiment was repeated three times to obtain the mean. Normally distributed datasets were analyzed by the unpaired Student's *t*-test for 2 independent groups or paired *t*-test for 2 dependent groups and the one-way analysis of variance (ANOVA) followed by the post-Bonferroni's multiple comparison test for ≥3 groups. For all statistical comparisons, a value of *P* < 0.05 was considered statistically significant and denoted with one, two, and three asterisks when lower than 0.05, 0.01, and 0.001, respectively.

## 3. Results

### 3.1. Macrophage Infiltration and VSMC Apoptosis Increase in the Aortic Media of Sm22*α*^−/−^ Mice with Ang II Infusion

**Figure 1:**
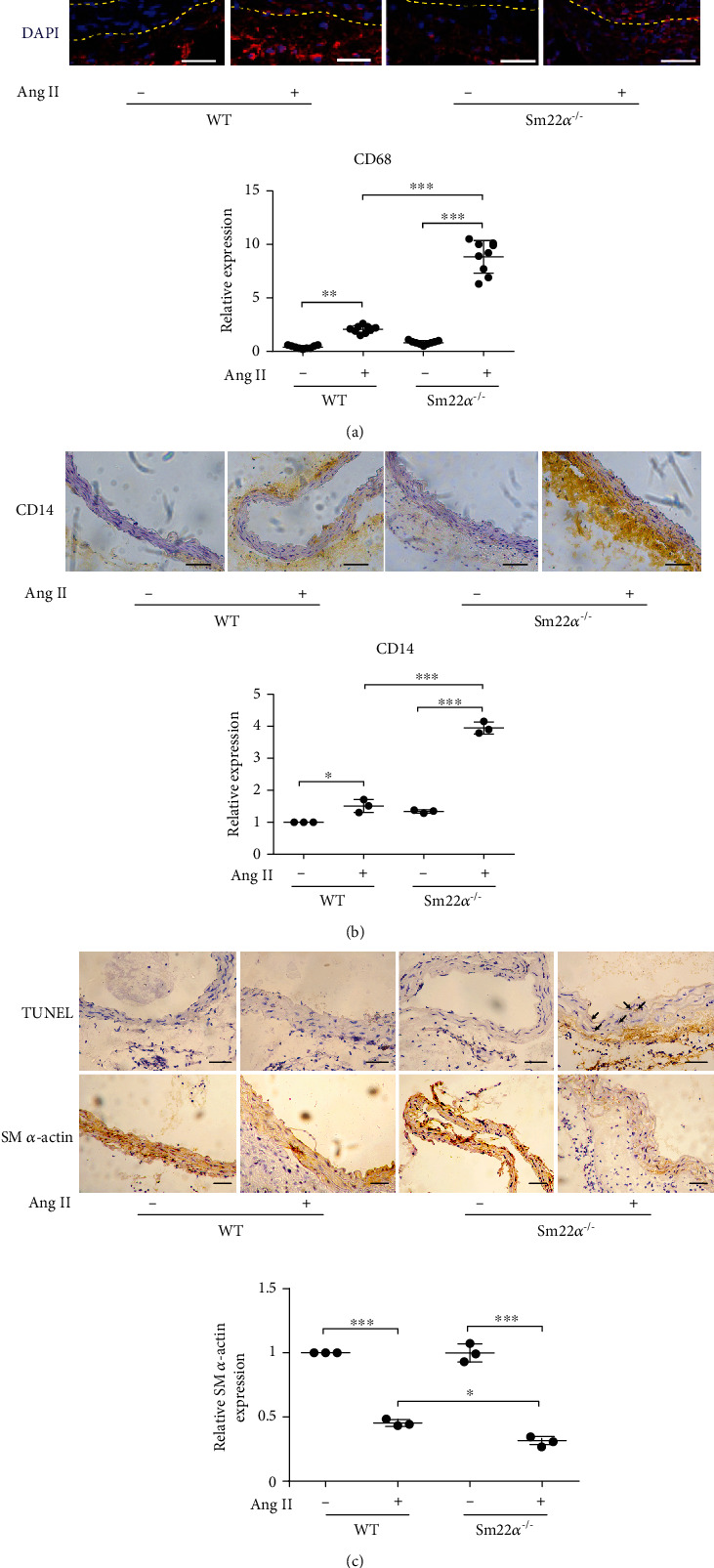
Macrophage infiltration and VSMC apoptosis increase in the aortic media of *Sm22α*^−/−^ mice with Ang II infusion. All mice were infused with saline (Ang II-) or Ang II (Ang II+) for 4 weeks. (a, b) Representative immunostaining of CD68 and CD14 in the abdominal aortas from WT or *Sm22α^−/−^* mice. Bars: 50 *μ*m (a) or 100 *μ*m (b). (c) Representative immunohistochemical staining of TUNEL and SM *α*-actin in abdominal aortas of WT and *Sm22α^−/−^* mice. Data are presented as mean ± SD of three independent experiments. ^∗^*P* < 0.05, ^∗∗^*P* < 0.01, and ^∗∗∗^*P* < 0.001.

We first verified that there was higher incidence of AAA formation and aggravated aortic macrophage infiltration in *Sm22α^−/−^* mice infused with Ang II compared to WT mice (Figures 1(a) and 1(b)), consistent with previous findings [10]. We next performed the TUNEL assay and showed that TUNEL-positive cells significantly increased in the aortic media of *Sm22α^−/−^* mice with Ang II infusion, compared with WT control, in accordance with the decreased number of medial VSMCs with SM *α*-actin-positive staining (Figure 1(c)). Thus, we speculated that SM22*α* loss may cause macrophage infiltration, associated with VSMC apoptosis.

### 3.2. SM22*α* Loss Precipitates Interaction of VSMCs with Macrophages via Expression of VCAM-1

**Figure 2:**
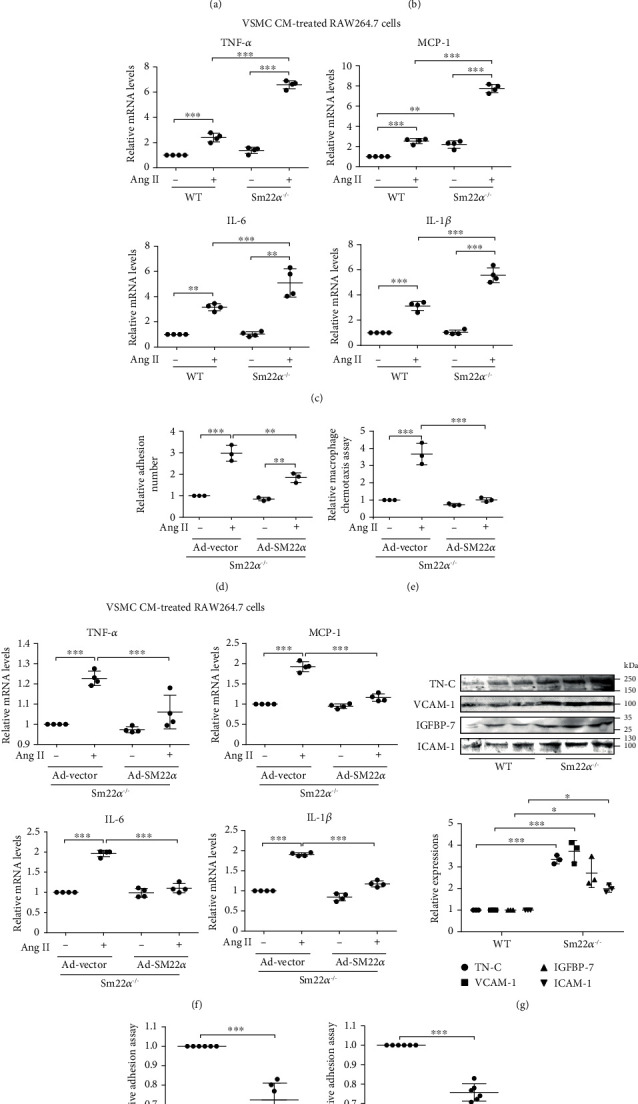
SM22*α* loss contributes to interaction of VSMCs with macrophages via expression of VCAM-1. (a) Relative quantification for RAW264.7 cell migration induced by the CM from WT and *Sm22α^−/−^* VSMCs treated with (+) or without (-) Ang II. (b) The fluorescent intensity quantification of calcein-AM-labeled RAW264.7 cell adhesion to WT or *Sm22α^−/−^* VSMCs. (c) qRT-PCR of the mRNA of TNF-*α*, MCP-1, IL-6, and IL-1*β* in RAW264.7 cells stimulated by CM from WT or *Sm22α^−/−^* VSMCs. (d) The relative number of RAW264.7 cell adhesion to Ad-vector- or Ad-SM22*α*-infected *Sm22α^−/−^* VSMCs. (e) Relative quantification for RAW264.7 cell migration induced by the CM from Ad-vector- or Ad-SM22*α*-infected *Sm22α^−/−^* VSMCs. (f) qRT-PCR of the mRNA of TNF-*α*, MCP-1, IL-6, and IL-1*β* in RAW264.7 cells stimulated by CM from Ad-vector- or Ad-SM22*α*-infected *Sm22α^−/−^* VSMCs. (g) Western blot analysis of differentially expressed adhesion molecules in CM from WT or *Sm22α^−/−^* VSMCs. (h, i) The fluorescent intensity quantification of calcein-AM-labeled RAW264.7 cell adhesion to *Sm22α^−/−^* VSMCs transfected with si-VCAM-1 (h) or preincubated with IgG or VCAM-1 neutralizing antibody (i). Data are presented as mean ± SD of three independent experiments. ^∗^*P* < 0.05, ^∗∗^*P* < 0.01, and ^∗∗∗^*P* < 0.001.

To assess a potential causative link between SM22*α* loss and macrophage infiltration observed from the *in vivo* study, we performed transwell migration assay using the Boyden chamber. *Sm22α^−/−^* VSMCs treated with or without Ang II treatment markedly induced the transwell migration of RAW264.7 cells (Figure 2(a)) and enhanced their interaction with RAW264.7 cells (Figure 2(b)). Furthermore, the expression of proinflammatory molecules TNF-*α*, MCP-1, IL-6, and IL-1*β* significantly increased in RAW264.7 cells treated with the conditional media (CM) of Ang II-induced *Sm22α^−/−^* VSMCs (Figure 2(c)). Rescue of SM22*α* expression reduced the interaction of *Sm22α^−/−^* VSMCs with macrophages (Figure 2(d)), which displayed reduced transwell migration (Figure 2(e)) and expression of proinflammatory molecules in RAW264.7 cells under the same conditions (Figure 2(f)), suggesting that *Sm22α*^−/−^ VSMCs are able to recruit and activate macrophages as SM22*α* was not expressed in WT mouse peritoneal macrophages and RAW264.7 cells (data not shown).

To explore how *Sm22α*^−/−^ VSMCs recruit and activate macrophages, we analyzed the complete secretome for the conditional media (CM) of VSMCs from wild-type (WT) and *Sm22α^−/−^* mice. Putative differentially expressed proteins generated by iTRAQ were identified (1.5-fold change). Using these criteria, there were a total of 267 proteins differentially expressed between *Sm22α*^−/−^ and WT mice. GO biological process (BP) revealed that the molecules related to cell adhesion, TN-C, VCAM-1, and NID-2 were significantly upregulated more than 20-fold in *Sm22α^−/−^* VSMCs (Supplementary Table 2). The expression of these adhesion molecules was verified by Western blot and greatly elevated in *Sm22α^−/−^* VSMCs compared with WT cells (Figure 2(g)), indicating that *Sm22α^−/−^* VSMCs are of proinflammatory secretory phenotype. The previous study has shown that VSMCs and macrophages are in direct contact in human atherosclerotic plaques by the expression of VCAM-1 [19]. To further validate that VCAM-1 secreted by *Sm22α^−/−^* VSMCs mediate macrophage infiltration, the specific siRNAs were used to knockdown the expression of the adhesion molecules. We found that knockdown of VCAM-1 markedly decreased the interaction between the VSMCs and RAW264.7 cells (Figure 2(h)). Similarly, the VCAM-1 neutralizing antibody removed this interaction (Figure 2(i)). These findings indicated that SM22*α* loss precipitates interaction of VSMCs with macrophages via expression of VCAM-1.

### 3.3. Macrophage-Derived circRasGEF1B Induces VSMC Apoptosis

**Figure 3:**
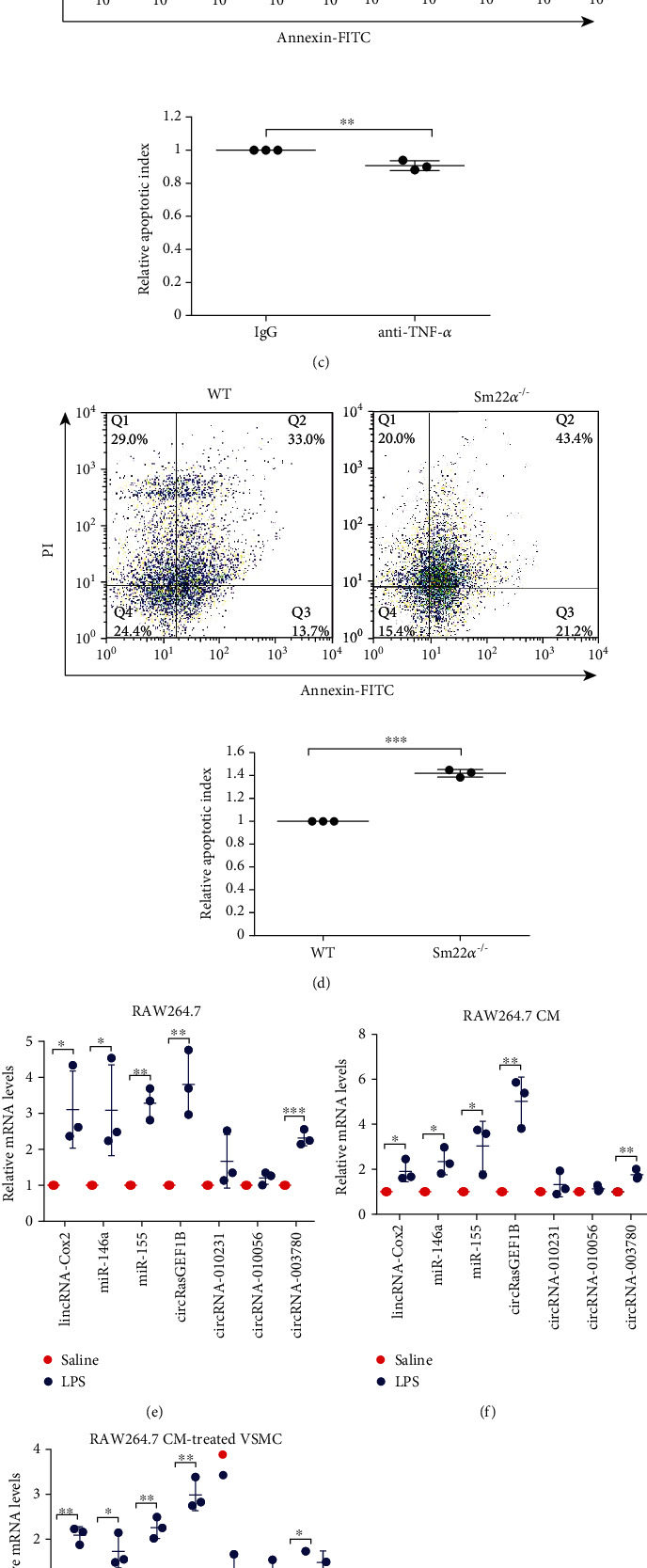
Macrophage-derived circRasGEF1B induces VSMC apoptosis. (a, b) Flow cytometry analysis (a) or Western blot (b) of cell apoptosis in MOVAS cells, a mouse VSMC line, pretreated with the CM from saline- or LPS-stimulated RAW264.7 cells. (c, d) Flow cytometry analysis of cell apoptosis in MOVAS cells pretreated with IgG or TNF-*α* neutralizing antibody (c) or in WT and *Sm22α^−/−^* VSMCs (d) treated with Ang II for 24 h. (e–g) qRT-PCR of lincRNA-Cox2, miR-146a, miR-155, circRasGEF1B, circRNA-010231, circRNA-010056, and circRNA-003780 in RAW264.7 cells (e), in the conditional media from RAW264.7 cells (f), and in MOVAS cells pretreated with the CM from RAW264.7 cells (g). (h) qRT-PCR of circRasGEF1B in RAW264.7 and MOVAS cells. (i) Confocal FISH images of circRasGEF1B in VSMCs pretreated with CM from RAW264.7 cell or not. Bars: 10 *μ*m. (j) RT-PCR of circRasGEF1B in MOVAS cells transfected with the pLCDH empty vector or pLCDH-circRasGEF1B plasmid. (k, l) TUNEL assay (k) and Western blot (l) of Bax and Bcl-2 in MOVAS cells transfected with the pLCDH empty vector or pLCDH-circRasGEF1B plasmid. Bars: 100 *μ*m (k). (m) TUNEL assay of cell apoptosis in WT or *Sm22α^−/−^* VSMCs transfected with the pLCDH-circRasGEF1B plasmid. Bars: 100 *μ*m. Data are presented as mean ± SD of three independent experiments. ^∗^*P* < 0.05, ^∗∗^*P* < 0.01, and ^∗∗∗^*P* < 0.001.

Human macrophages potently induce VSMC apoptosis via direct cell-cell interactions mediated by Fas/Fas-L [20]. We showed that the activity of apoptosis was increased by 1.62-fold in VSMCs treated with the CM from lipopolysaccharide- (LPS-) activated RAW264.7 cells compared to those treated with the control CM (Figure 3(a)), accompanied by increased expression of Bax and cleaved caspase-3 and decreased Bcl-2 protein (Figure 3(b)). To eliminate the effect of TNF-*α* from LPS-activated macrophages on apoptosis, VSMCs were induced by the RAW264.7 CM treated with the TNF-*α* neutralizing antibody. We showed that removing TNF-*α* did not abolish the RAW264.7 CM-induced apoptosis of VSMCs (Figure 3(c)). Furthermore, the apoptosis of *Sm22α^−/−^* VSMCs was higher than that of WT cells (Figure 3(d)).

It has been known that exogenous transcripts reprogram recipient cell gene expression and function [21–25]. To examine potential mediators for induction of VSMC apoptosis by macrophages, we screened and identified a set of noncoding RNAs (ncRNAs) highly expressed in activated macrophages, including lincRNA-Cox2, miR-146a, miR-155, circRasGEF1B, circRNA-010231, circRNA-010056, and circRNA-003780 [21, 26–30]. Among them, the expression of five noncoding RNAs increased in the activated RAW264.7 cells and their CM; in particular, increase in circRasGEF1B was more much (Figures 3(e) and 3(f)). Although all of these noncoding RNAs were detected, the level of circRasGEF1B was the highest in VSMCs treated with the CM of activated RAW264.7 cells (Figure 3(g)). Furthermore, the expression of circRasGEF1B was specific to the macrophage as it was low and unchanged in VSMCs upon LPS treatment (Figure 3(h)). This result was further confirmed by the fluorescence *in situ* hybridization (FISH) assay. Fluorescence-stained circRasGEF1B was observed only in the cytoplasm of VSMCs treated with the CM of activated RAW264.7 cells (Figure 3(i)), suggesting that circRasGEF1B is delivered from the macrophages to VSMCs.

To further determine that macrophage-derived circRasGEF1B triggers VSMC apoptosis, VSMCs were transfected with the pLCDH-circRasGEF1B plasmid. The percentage of TUNEL-stained cells and the ratio of Bax/Bcl-2 were increased in VSMCs overexpressing circRasGEF1B (Figures 3(j) and 3(k)). circRasGEF1B-mediated apoptosis was more serious in *Sm22α*^−/−^ VSMCs than in WT cells (Figure 3(l)). These data suggest that circRasGEF1B is a new mediator for macrophages inducing VSMC apoptosis.

### 3.4. ZFP36 Mediates circRasGEF1B-Induced Apoptotic Programming of VSMCs

**Figure 4:**
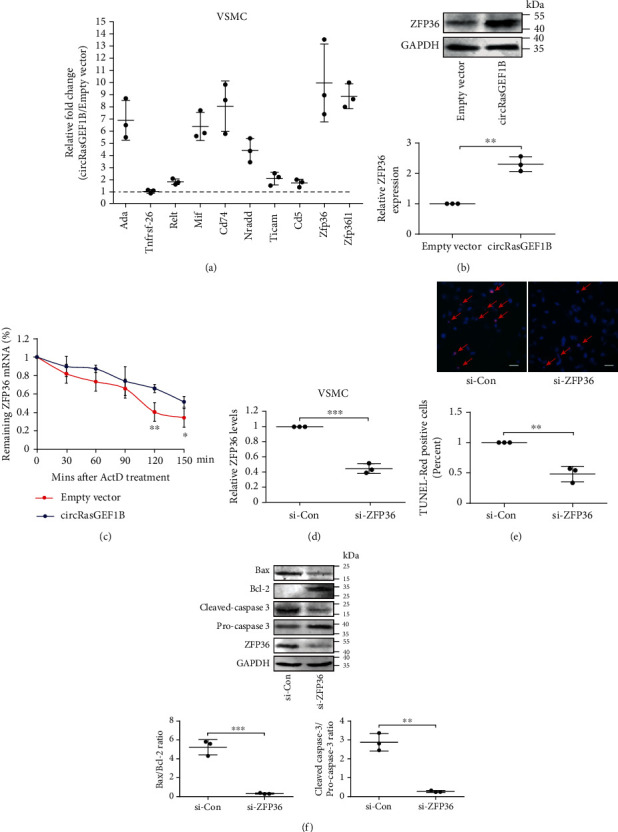
ZFP36 mediates circRasGEF1B-induced apoptotic programming of VSMCs. (a) The relative fold change of the mRNA of Ada, Tnfrsf-26, Relt, Mif, Cd74, Nradd, Ticam1, Cd5, Zfp36, and Zfp36l1 in MOVAS cells transfected with the pLCDH-circRasGEF1B plasmid compared to the pLCDH empty vector group. (b) Western blot and densitometric analysis of the expression of ZFP36 in MOVAS cells transfected with the pLCDH empty vector or pLCDH-circRasGEF1B plasmid. (c) qRT-PCR of remaining ZFP36 mRNA in pLCDH empty vector- or pLCDH-circRasGEF1B plasmid-transfected MOVAS cells stimulated by ActD stimulation for indicated time. Values represent mean ± SD from 3 independent experiments; ^∗^*P* < 0.05, ^∗∗^*P* < 0.01 vs. empty vector group at the same time point. (d) qRT-PCR of ZFP36 mRNA in MOVAS cells transfected with si-Con or si-ZFP36. (e, f) TUNEL assay (e) or Western blot (f) for cell apoptosis in circRasGEF1B-overexpressed MOVAS cells transfected with si-Con or si-ZFP36. Data are presented as mean ± SD of three independent experiments. ^∗^*P* < 0.05, ^∗∗^*P* < 0.01, and ^∗∗∗^*P* < 0.001.

The transcriptome-wide data in control and circRasGEF1B-deficient macrophages have been reported using RNA sequencing (RNA-seq) [13]. Based on these data, we identified putative differentially expressed genes (2-fold change cut-off) through high-throughput transcriptomic analysis. Of the differentially expressed genes, 10 potential apoptosis-related genes were screened, including Ada, Tnfrsf-26, Relt, Mif, Cd74, Nradd, Ticam1, Cd5, Zfp36, and Zfp36l1 (Supplementary Table 3), and all of them were downregulated in the circRasGEF1B-deficient group. To determine that circRasGEF1B regulates the expression of these genes, we tested the expression of these 10 genes in pLCDH-circRasGEF1B-transfected VSMCs using qRT-PCR. We showed that ZFP36 mRNA level was obviously upregulated following circRasGEF1B overexpression (Figure 4(a)), accompanied by increased ZFP36 protein (Figure 4(b)). To determine how circRasGEF1B upregulates ZFP36 expression, we measured ZFP36 mRNA half-life after blocking de novo RNA synthesis with ActD in VSMCs. The half-life of ZFP36 mRNA was increased in circRasGEF1B-overexpressed VSMCs compared with the control group (Figure 4(c)), indicating that the stabilization of ZFP36 mRNA was enhanced. To confirm whether ZFP36 is associated with circRasGEF1B-induced VSMC apoptosis, we silenced ZFP36 expression by using specific siRNAs in circRasGEF1B-overexpressed VSMCs (Figure 4(d)) and showed that knockdown of ZFP36 abolished circRasGEF1B-induced apoptosis (Figure 4(e)), accompanied by a decreased Bax/Bcl-2 ratio and cleaved caspase-3 expression (Figure 4(f)), suggesting that ZFP36 mediates circRasGEF1B-induced apoptosis of VSMCs.

### 3.5. circRasGEF1B Guides ZFP36 to Preferentially Bind to and Decay Bcl-2 mRNA in VSMCs

**Figure 5:**
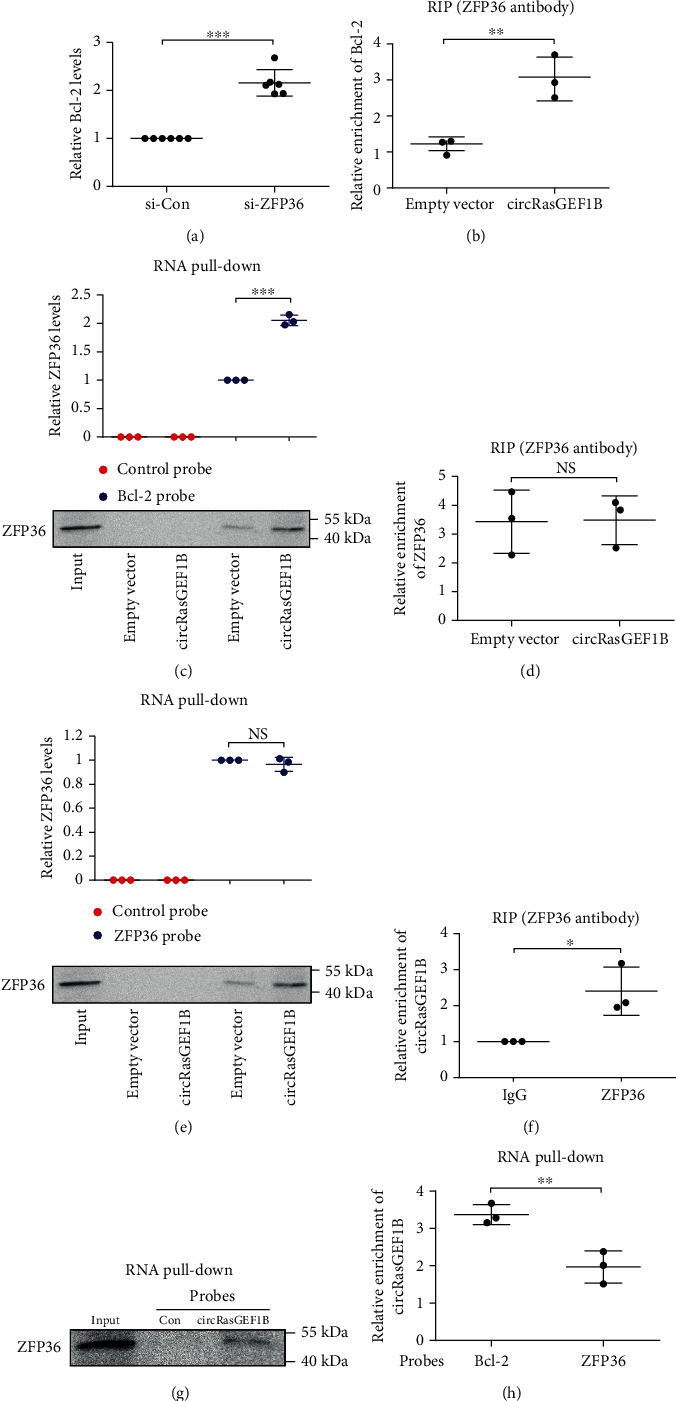
circRasGEF1B guides ZFP36 to preferentially bind to and decay Bcl-2 mRNA in VSMCs. (a) qRT-PCR of Bcl-2 mRNA levels in circRasGEF1B-overexpressed MOVAS cells transfected with si-Con or si-ZFP36. (b) RIP assay of Bcl-2 mRNAs retrieved by using a ZFP36 antibody in MOVAS cells transfected with the pLCDH empty vector or pLCDH-circRasGEF1B plasmid. (c) RNA pull-down assay of ZFP36 protein retrieved by Bcl-2 mRNA probes in MOVAS cells transfected with the pLCDH empty vector or pLCDH-circRasGEF1B plasmid. (d) RIP assay of ZFP36 mRNAs retrieved by using a ZFP36 antibody in MOVAS cells transfected with the pLCDH empty vector or pLCDH-circRasGEF1B plasmid. (e) RNA pull-down assay of ZFP36 proteins retrieved by using ZFP36 mRNA probes in MOVAS cells transfected with the pLCDH empty vector or pLCDH-circRasGEF1B plasmid. (f) RIP assay of circRasGEF1B levels retrieved by using an IgG or ZFP36 antibody in MOVAS cells transfected with the pLCDH-circRasGEF1B plasmid. (g) RNA pull-down assay of ZFP36 proteins retrieved by using a circRasGEF1B probe in MOVAS cells transfected with the pLCDH-circRasGEF1B plasmid. (h) RNA pull-down assay of circRasGEF1B levels retrieved by using Bcl-2 or ZFP36 mRNA probes in MOVAS cells transfected with the pLCDH-circRasGEF1B plasmid. Data are presented as mean ± SD of three independent experiments. ^∗^*P* < 0.05, ^∗∗^*P* < 0.01, and ^∗∗∗^*P* < 0.001.

It has been reported that the ZFP36 family promotes mRNA decay via binding to the 3′-UTRs of their target mRNAs with AU-rich element (ARE) to maintain appropriate target transcript and protein levels, including Bcl-2 and ZFP36 itself [31]. As mentioned above, overexpression of circRasGEF1B reduced Bcl-2 expression (Figure 3(l)). To verify the causal relationship between increased ZFP36 and decreased Bcl-2 level, we knocked down ZFP36 and showed increased level of Bcl-2 mRNA in circRasGEF1B-overexpressed VSMCs (Figure 5(a)). To examine whether ZFP36 interacts with Bcl-2 mRNA, we performed RIP using an anti-ZFP36 antibody and RNA pull-down using a Bcl-2 mRNA probe, respectively. We found that the interaction between ZFP36 protein and Bcl-2 mRNA was increased in circRasGEF1B-transfected VSMCs (Figures 5(b) and 5(c)). However, overexpression of circRasGEF1B did not increase the interaction of ZFP36 protein with its mRNA that contains AU-rich element (Figures 5(d) and 5(e)).

To ascertain the mechanism by which ZFP36 preferentially binds to and decays Bcl-2 mRNA in circRasGEF1B-overexpressed VSMCs, we first predicted the potential RNA region for circRasGEF1B binding to the two mRNAs and ZFP36 protein using the RegRNA 2.0 [32] and catRAPID program [33], respectively, and assessed the hybridization Δ*G* values for RNA-RNA pairs by RNAup Server [34]. The potential binding region of ZFP36 protein was observed in the circRasGEF1B sequence, and there were obviously lower Δ*G* values between circRasGEF1B and Bcl-2 mRNA, compared with binding to ZFP36 mRNA (Supplementary Tables 4 and 5); namely, circRasGEF1B may serve to bind both ZFP36 and Bcl-2 mRNAs together. This let us to further explore the mechanism by which circRasGEF1B directs ZFP36 to preferentially bind to Bcl-2 mRNA in the presence of ZFP36 mRNA. RIP and RNA pull-down assay showed that circRasGEF1B was retrieved by using a ZFP36 antibody, and ZFP36 proteins were also retrieved by using a circRasGEF1B probe in VSMCs transfected with the pLCDH-circRasGEF1B plasmid (Figures 5(f) and 5(g)). Compared with the ZFP36 mRNA probe, the Bcl-2 mRNA probe retrieved more circRasGEF1B (Figure 5(h)). Thus, circRasGEF1B enables ZFP36 to preferentially bind to Bcl-2 mRNA in the presence of ZFP36 mRNA.

### 3.6. circRasGEF1B Directly Interacts with Both ZFP36 and Bcl-2 mRNAs in a Sequence-Specific Manner

**Figure 6:**
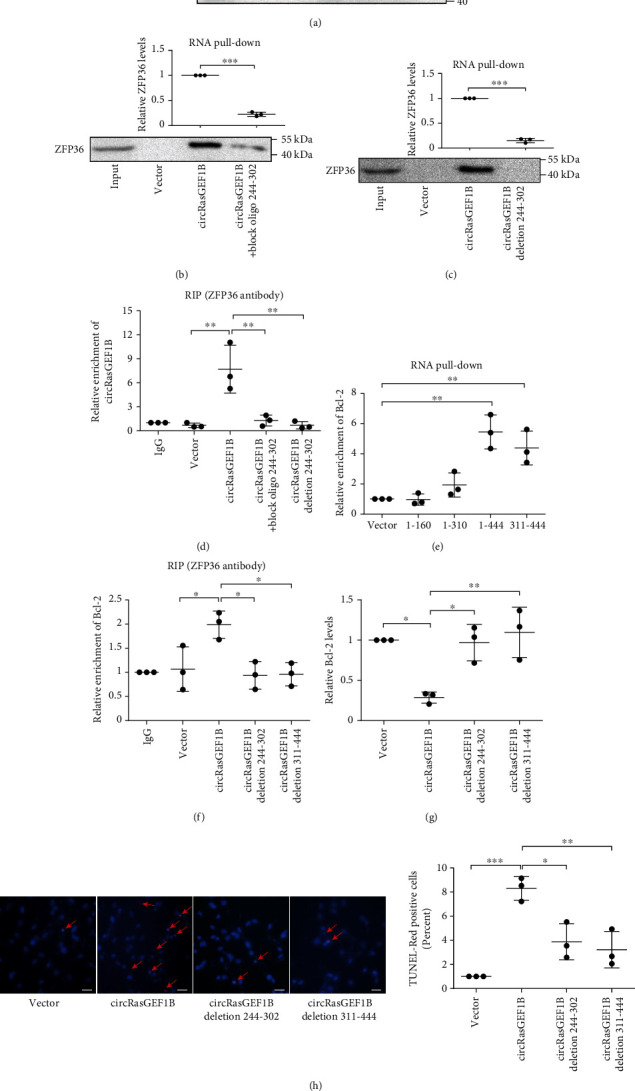
circRasGEF1B directly interacts with both ZFP36 and Bcl-2 mRNAs in a sequence-specific manner. (a) RNA pull-down assay of ZFP36 proteins retrieved by using probes of circRasGEF1B deletion mutants in MOVAS cells transfected with circRasGEF1B deletion mutants. (b, c) RNA pull-down assay of ZFP36 proteins retrieved by using circRasGEF1B probes in MOVAS cells transfected with circRasGEF1B, block oligo 244-302 (b), or 244-302 deletion mutant (c). (d) RIP assay of circRasGEF1B retrieved by using a ZFP36 antibody in MOVAS cells transfected with circRasGEF1B, block oligo 244-302, or 244-302 deletion mutant. (e) RNA pull-down assay of Bcl-2 mRNA retrieved by using probes of circRasGEF1B deletion mutants in MOVAS cells transfected with circRasGEF1B deletion mutants. (f) RIP assay of Bcl-2 mRNA retrieved by using a ZFP36 antibody in MOVAS cells transfected with circRasGEF1B or deletion mutants. (g, h) qRT-PCR of Bcl-2 mRNA levels (g) or TUNEL assay of cell apoptosis (h) in MOVAS cells transfected with circRasGEF1B or deletion mutants. Bars: 100 *μ*m (h). Data are presented as mean ± SD of three independent experiments. ^∗^*P* < 0.05, ^∗∗^*P* < 0.01, and ^∗∗∗^*P* < 0.001.

To identify the binding sites of ZFP36 in the circRasGEF1B sequence, a series of circRasGEF1B deletion mutants were used to determine the regions in circRasGEF1B that binds to ZFP36. We showed that the mutants retaining the nt 161-310 sequence of circRasGEF1B bound to ZFP36, whereas other mutants completely lost their binding capacity (Figure 6(a)). Additionally, the catRAPID predicted the nt 244-302 motif of circRasGEF1B is a binding site for ZFP36. To verify the prediction, blocking oligo that was complimentary to the ZFP36 binding sites in the circRasGEF1B sequence was transfected into VSMCs. We showed that the blocking oligo inhibited the interaction of circRasGEF1B with ZFP36 in the RNA pull-down assay (Figure 6(b)). Furthermore, the interaction of circRasGEF1B with ZFP36 was enhanced in VSMCs transfected with circRasGEF1B but not the circRasGEF1B deletion mutant in RNA pull-down and RIP assays (Figures 6(c) and 6(d)).

As mentioned above, there were lower hybridization Δ*G* values between circRasGEF1B and Bcl-2 mRNA, compared with binding to ZFP36 mRNA (Supplementary Tables 4 and 5). To further confirm the binding sequences for Bcl-2 mRNA in circRasGEF1B, a series of biotinylated DNA probes were synthesized and incubated with VSMCs transfected with different circRasGEF1B deletion mutants, respectively. The mixture was subsequently pulled down with streptavidin beads, followed by real-time PCR. We showed that the circRasGEF1B mutants retaining nt 311-444 bound to Bcl-2 mRNA (Figure 6(e)). In contrast, the mutants of circRasGEF1B deleting nt 244-302 or 311-444 were unable to recruit ZFP36 to decay Bcl-2 mRNA (Figure 6(f)), which resulted in reduced VSMC apoptosis (Figures 6(g) and 6(h)). These data suggest that circRasGEF1B, as a platform, recruits ZFP36 to selectively decay Bcl-2 mRNAs and may be a critical determinant of ZFP36-mRNA target specificity.

## 4. Discussion

**Figure 7:**
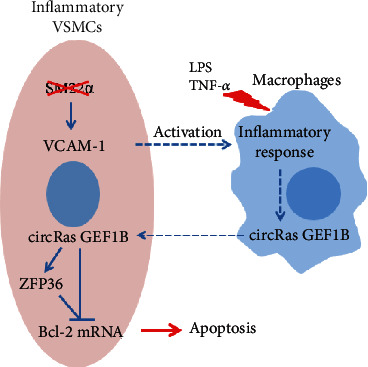
Mechanism of SM22*α* ameliorates macrophage-induced apoptosis in VSMCs. VSMCs missing SM22*α* activate macrophages in a VCAM-1-dependent manner and induce the self-apoptosis via circRasGEF1B-ZFP36-mediated Bcl-2 mRNA decay.

In the current study, we demonstrated that *Sm22α*^−/−^ VSMCs favor interacting with macrophages and displayed higher sensitivity to apoptosis (Figure 7). Our findings highlight that (1) VSMCs missing SM22*α* are able to recruit and activate macrophages in a VCAM-1-dependent manner, creating an inflammatory microenvironment; (2) macrophage-derived circRasGEF1B reprograms VSMC apoptosis via recruiting ZFP36 to selectively bind to and decay Bcl-2 mRNA; and (3) the circRasGEF1B-ZFP36 axis is a novel pathway for communication between macrophages and VSMCs and a new mechanism by which macrophages determine VSMC fate. Thus, perhaps modulating VSMC phenotypes to a differentiated or reparative state by targeting SM22*α* or circRasGEF1B reduces the harmful communication between macrophages and VSMCs and may be beneficial for therapies of aortic aneurysm and its clinical complications.

SM22*α* has been considered to be one of the hallmarks of SMC phenotypic switching [35, 36]. Our recent studies have documented that SM22*α* is vital in maintaining VSMC contractile phenotype and vascular homeostasis [37] and is downregulated by endothelial injury or renin-angiotensin system activation, contributing to proliferation and hypertrophy of VSMCs [6, 38]. VSMCs missing SM22*α* may provide a vascular environment susceptible to inflammation and predispose the aorta to aneurismal formation [10]. However, the role of SM22*α* in VSMC apoptosis remains to be not fully studied. Herein, we found that disruption of SM22*α* significantly increased the expression and secretion of VCAM-1 and led to macrophage recruitment *in vivo* and *in vitro* under Ang II treatment, which was abolished by rescued expression of SM22*α* or by VCAM-1 neutralizing antibody. Decrease in SM22*α* expression has been well defined in a variety of VSMC-driven vascular diseases. Our and other studies provide further evidence of the key role of SM22*α* in maintaining vascular structural integrity and the pathophysiology of multiple vascular diseases not just as a biomarker of contractile SMC. Although the recent study considered that the effect of SM22*α* deficiency on AAA formation was not mediated by increasing cell apoptosis [10], this conclusion was only based on the aortic cleaved caspase-3 expression by Western blotting in *ApoE*^−/−^ mouse *in vivo* study and ignored the effect of *ApoE* deficiency on VSMC apoptosis. Ang II-infused *ApoE*^−/−^mice, as a popular mouse model for aneurysm research, displaying vascular matrix degradation and inflammation can be far more than the changes observed in *Sm22α*^−/−^ mice under the same conditions, and the effect of AAV-SM22*α in vivo* could be not enough to ameliorate these lesions in *ApoE*^−/−^mice.

Involvement of macrophages in the pathogenesis of unstable plaque and aortic aneurysm has been well defined in the past decade. Human macrophages potently induce VSMC apoptosis via direct cell-cell interactions mediated by Fas/Fas-L, promoting plaque rupture [20]. Our present study provided evidence that macrophages induced VSMC apoptosis by a circRNA-mediated mechanism. We identified a set of noncoding RNAs highly expressed in LPS-activated RAW264.7 cells and validated that the expression of circRasGEF1B was highest among them and transferred into VSMCs, associated with increased apoptosis. Although the expression of TNF-*α* that is a proapoptotic cytokine was induced in the activated macrophages, we showed that the apoptosis of VSMCs was still higher upon treatment with the macrophage CM that was treated with the TNF-*α* neutralizing antibody. In contrast, the activity of apoptosis reduced in si-circRasGEF1B-treated cells that exhibited increase in Bcl-2 expression. Thus, circRasGEF1B is a novel mediator by which macrophages induce VSMC apoptosis.

Cytoplasmic mRNA decay constitutes an important posttranscriptional mechanism in mammalian cells. The regulation of cytoplasmic mRNA half-life is mediated by mRNA-binding proteins and noncoding RNAs (ncRNAs), such as microRNAs and long noncoding RNAs [39]. The AU-rich elements (AREs) are the largest group of cis-acting elements controlling mRNA decay. ZFP36, also known as tristetraprolin (TTP), is an ancient RNA-binding protein belonging to a CCCH tandem zinc finger protein family and plays a critical role in a wide variety of physiological processes through maintaining appropriate target transcript and protein levels as part of normal cell and tissue homeostasis by regulating the expression of ARE-containing mRNAs including its own [40, 41]. It has been reported that ZFP36 inhibits the expression of Bcl-2 and enhances cisplatin sensitivity of HNSCC cells [42]. We showed that overexpression of circRasGEF1B significantly increased ZFP36 expression at mRNA and protein levels with increased ZFP36 mRNA stability in VSMCs. Furthermore, knockdown of ZFP36 attenuated circRasGEF1B-induced apoptosis of VSMCs, suggesting that ZFP36 is a target for the effect circRasGEF1B on apoptosis in VSMCs. We further validated the interaction among circRasGEF1B, ZFP36, and Bcl-2 mRNA in a sequence-specific manner. circRasGEF1B, as a scaffold, recruited ZFP36 to bind to and decay Bcl-2 mRNA and promoted VSMC apoptosis. We considered that circRasGEF1B may play a key role for determining ZFP36-mRNA target specificity.

There are several limitations to this study. It has been known that activated macrophages release inflammatory factors to induce VSMC apoptosis via the membrane receptor pathway. Now, it is unknown how macrophage-derived circRasGEF1B and proapoptosis factor-activated pathways interact and converge to regulate Bcl-2 mRNA stability in VSMCs. Is it the same in macrophages? It is necessary to further investigate the crosstalking between this apoptosis pathway and other functions of circRasGEF1B.

In summary, we provide evidence that *Sm22α^−/−^* VSMCs favor interacting with macrophages and the resulting activation of the circRasGEF1B-ZFP36 axis is a novel mechanism underlying macrophages inducing VSMC apoptosis.

## Data Availability

The data used to support the findings of this study are available from the corresponding author upon request.

## Abbreviations

AAA: Abdominal aortic aneurysm
Ang II: Angiotensin II
ARE: AU-rich element
circRNA: Circular RNA
HE: Hematoxylin and eosin
ICAM-1: Intercellular cell adhesion molecule-1
LPS: Lipopolysaccharide
MCP-1: Monocyte chemotactic protein-1
RIP: RNA immunoprecipitation
RNA-seq: RNA sequencing
ROS: Reactive oxygen species
siRNA: Small interference RNA
SM22*α*: Smooth muscle 22*α*
TNF: Tumor necrosis factor
TUNEL: Terminal deoxynucleotidyl transferase-mediated dUTP nick end labeling
UTR: Untranslated region
VCAM-1: Vascular cell adhesion molecule-1
VSMC: Vascular smooth muscle cell
WT: Wild type.

## Supplementary Materials

Supplementary Materials Supplementary Table 1: PCR primer sequence. Supplementary Table 2: list of enriched (*P* < 0.05) differentially expressed proteins related to cellular adhesion identified by SM22*α* knockout in VSMC conditional media. Supplementary Table 3: list of enriched (*P* < 0.05) differentially expressed genes related to apoptosis identified by circRasGEF1B knockdown in RAW264.7 cells. Supplementary Table 4: predicted ncRNA hybridization regions of ZFP36 mRNA and Δ*G* value for circRasGEF1B-ZFP36 mRNA duplexes. Supplementary Table 5: predicted ncRNA hybridization regions of Bcl-2 mRNA and Δ*G* value for circRasGEF1B-Bcl-2 mRNA duplexes.
