# Supplementary material for: SM22α Loss Contributes to Apoptosis of Vascular Smooth Muscle Cells via Macrophage-Derived circRasGEF1B
Source: Oxid Med Cell Longev. 2021 Mar 16;2021:5564884. doi: 10.1155/2021/5564884 (PMC8026322; doi:10.1155/2021/5564884)
Supplement: Supplementary Materials — Supplementary Table 1: PCR primer sequence. Supplementary Table 2: list of enriched (P < 0.05) differentially expressed proteins related to cellular adhesion identified by SM22α knockout in VSMC conditional media. Supplementary Table 3: list of enriched (P < 0.05) differentially expressed genes related to apoptosis identified by circRasGEF1B knockdown in RAW264.7 cells. Supplementary Table 4: predicted ncRNA hybridization regions of ZFP36 mRNA and ΔG value for circRasGEF1B-ZFP36 mRNA duplexes. Supplementary Table 5: predicted ncRNA hybridization regions of Bcl-2 mRNA and ΔG value for circRasGEF1B-Bcl-2 mRNA duplexes. [file 5564884.f1.zip › R1-SUBMIT-Supplementary data-0301.docx]

**Supplementary data**

Supplementary Table 1: PCR primer sequence

| Genes | Sequences |
| --- | --- |
| VCAM-1 | Forward: 5’- AGTTGGGGATTCGGTTGTTCT -3’  Reverse: 5’- CCCCTCATTCCTTACCACCC -3’ |
| TNF-α | Forward: 5’- CCCTCACACTCAGATCATCTTCT -3’  Reverse: 5’- GCTACGACGTGGGCTACAG -3’ |
| IL-1β | Forward: 5’- GCAACTGTTCCTGAACTCAACT -3’  Reverse: 5′- ATCTTTTGGGGTCCGTCAACT -3’ |
| IL-6 | Forward: 5’- TAGTCCTTCCTACCCCAATTTCC -3’  Reverse: 5’- TTGGTCCTTAGCCACTCCTTC -3’ |
| MCP-1 | Forward: 5’- CTTCTGGGCCTGCTGTTCA -3’  Reverse: 5’- CCAGCCTACTCATTGGGATCA -3’ |
| Ada | Forward: 5’- ACCCGCATTCAACAAACCCA -3’  Reverse: 5’- AGGGCGATGCCTCTCTTCT -3’ |
| Tnfrsf-26 | Forward: 5’- TTCAAACATGAGAACCTCTGCTG -3’  Reverse: 5’- CACATGGAGCACATTCACTCT -3’ |
| Relt | Forward: 5’- CAGGGCCTGATGATGAAGCG -3’  Reverse: 5’- GGGGCAAGTTCTGCATAATGTG -3’ |
| Mif | Forward: 5’- GCCAGAGGGGTTTCTGTCG -3’  Reverse: 5’- GTTCGTGCCGCTAAAGTCA -3’ |
| Cd74 | Forward: 5’- AGTGCGACGAGAACGGTAAC -3’  Reverse: 5’- CGTTGGGGAACACACACCA -3’ |
| Nradd | Forward: 5’- CGGCTCCTGATGATGGGTG -3’  Reverse: 5’- GTATAGGCTGGCATTTGGTCAC -3’ |
| Ticam1 | Forward: 5’- AACCTCCACATCCCCTGTTTT -3’  Reverse: 5’- CGGGCACCTGAAATTCCTCA -3’ |
| Cd5 | Forward: 5’- GGTGATGCTAAGTGGCTCCAA -3’  Reverse: 5’- GGGGTCTGTTCAATGAAGGGAA -3’ |
| ZFP36 | Forward: 5’- CCACCTCCTCTCGATACAAGA -3’  Reverse: 5’- GCTTGGCGAAGTTCACCCA -3’ |
| ZFP36l1 | Forward: 5’- GCTTTCGAGACCGCTCTTTCT -3’  Reverse: 5’- TTGTCCCCGTACTTACAGGCA -3’ |
| lincRNA-Cox2 | Forward: 5’-AAGGAAGCTTGGCGTTGTGA -3’  Reverse: 5’-GAGAGGTGAGGAGTCTTATG -3’ |
| miR-146a | Forward: 5’-ACCAGCAGTCCTCTTGATGC -3’  Reverse: 5’-GACGAGCTGCTTCAAGTTCC -3’ |
| miR-155 | Forward: 5’- GGGGGTTAATGCTAATTGTGAT -3’  Reverse: 5’- AGTGCGTGTCGTGG -3’ |
| U6 | Forward: 5’- GCTTCGGCAGCACATATACTAAAAT -3’；  Reverse: 5’- CGCTTCACGAATTTGCGTGTCAT -3’； |
| circRasGEF1B | Forward: 5’- GTATGACTTCCGGGACGAGA -3’  Reverse: 5’- TGTTGGATAAGGGCTTCCAG -3’ |
| circRNA-010231 | Forward: 5’- TTGAGGCGAATGGCTGAG -3’  Reverse: 5’- GCGGGAGGCTTGAATGTC -3’ |
| circRNA-010056 | Forward: 5’- TCACCAGGAGAATCCCAGTC -3’  Reverse: 5’- GAACTCTAAAATCAGGCT -3’ |
| circRNA-003780 | Forward: 5’- AGTGCCTCAGGTTTCTGG -3’；  Reverse: 5’- ATTCTGTCTTCCTTTCTTGC -3’ |
| Bcl-2 | Forward: 5’- GTCGCTACCGTCGTGACTTC -3’  Reverse: 5’- CAGACATGCACCTACCCAGC -3’ |
| GAPDH | Forward: 5’- AGGTCGGTGTGAACGGATTTG -3’  Reverse: 5’- TGTAGACCATGTAGTTGAGGTCA -3’ |
| β-actin | Forward: 5’- GGCTGTATTCCCCTCCATCG -3’  Reverse: 5’- CCAGTTGGTAACAATGCCATGT -3’ |

Supplementary Table 2: List of enriched (*p* < 0.05) differentially expressed proteins related to cellular adhesion identified by SM22α-knockout in VSMC conditional media.

| Differentially protein name | FDR | Expression ratio of Sm22α^-/-^: WT |
| --- | --- | --- |
| Tenascin C (TN-C) | 5.23452E-11 | 28.054 |
| Vascular cell adhesion molecule-1 (VCAM-1) | 1E-16 | 25.586 |
| Nidogen-2 (NID-2) | 1E-16 | 23.988 |
| Immunoglobulin superfamily containing leucine-rich repeat protein (ISLR) | 0.000111944 | 21.086 |
| Insulin-like growth factor binding protein-7 (IGFBP-7) | 0.000101766 | 16.144 |
| Cadherin-11 (CDH-11) | 4.84032E-06 | 15.276 |
| Tubulointerstitial nephritis antigen-like-1 (TINAGL-1) | 0.000693368 | 13.428 |
| Collagen, type XII, alpha-1 (COL12A-1) | 1E-16 | 12.706 |
| Nidogen-1 (NID-1) | 2.60696E-11 | 11.272 |
| Intercellular adhesion molecule-1 (ICAM-1) | 6.68351E-05 | 5.546 |
| Neogenin-1 (NEO-1) | 0.000970632 | 5.495 |
| Ephrin B-2 (EFNB-2) | 0.01338372 | 5.012 |
| MCG133388, isoform CRA_f (PCDHGC-5) | 0.006483611 | 3.631 |
| Elastin microfibril interface-2 (EMILIN-2) | 0.006827088 | 3.597 |
| Collagen, type XIV, alpha-1 (COL14A-1) | 0.000564898 | 3.342 |
| Collagen, type V, alpha-1 (COL5A-1) | 9.87026E-07 | 3.105 |
| Laminin, alpha-4 (LAMA-4) | 0.000379543 | 2.965 |
| Disintegrin and metalloproteinase domain-containing protein 15 (ADAM-15) | 0.000127728 | 2.729 |
| Lectin, galactoside-binding, soluble, 3 binding protein (LGALS3BP) | 0.02344035 | 2.312 |
| Collagen, type VI, alpha-1 (COL6A-1) | 7.0075E-10 | 2.208 |
| Neuroplastin (NPTN) | 0.029002421 | 1.959 |
| Thrombospondin-1 (THBS-1) | 0.000167497 | 0.449 |
| Talin-1 (TLN-1) | 0.027219711 | 0.449 |
| Fibronectin leucine rich transmembrane protein-2 (FLRT-2) | 0.043631971 | 0.229 |
| Periostin, osteoblast specific factor (POSTN) | 0.000526394 | 0.180 |
| Cell adhesion molecule-4 (CADM-4) | 0.04224835 | 0.156 |
| Contactin-2 (CNTN-2) | 1.10498E-11 | 0.127 |
| Neural cell adhesion molecule-1 (NCAM-1) | 2.67716E-08 | 0.127 |
| Cadherin-15 (CDH-15) | 4.15455E-08 | 0.083 |

Supplementary Table 3: List of enriched (*p* < 0.05) differentially expressed genes related to apoptosis identified by circRasGEF1B-knockdown in RAW264.7 cells.

| Differentially gene name | FDR | Expression ratio of  Control: circRasGEF1B-knockdown |
| --- | --- | --- |
| Ada | 0.000164312 | 4.239 |
| Tnfrsf-26 | 5.05E-17 | 8.878 |
| Relt | 0.006040954 | 3.208 |
| Mif | 4.40E-12 | 7.420 |
| Cd74 | 5.43E-13 | 5.944 |
| Nradd | 0.003355469 | 3.399 |
| Ticam1 | 1.44E-09 | 6.548 |
| Cd5 | 7.34E-08 | 5.872 |
| Zfp36 | 1.93E-09 | 6.499 |
| Zfp36l1 | 1.14E-06 | 5.351 |

Supplementary Table 4: Predicted ncRNA hybridization regions of ZFP36 mRNA and △G value for circRasGEF1B-ZFP36 mRNA duplexes

| ncRNA hybridization regions (nt) | Sequences | △G (kcal/mol) |
| --- | --- | --- |
| 1251-1301 | UCUUCUGUUGUUUUUGAGAUAGGAGCUUAUUAUGGUACCCCAGGCUGGCUU | -9.50 |
| 1255-1297 | CUGUUGUUUUUGAGAUAGGAGCUUAUUAUGGUACCCCAGGCUG | -10.31 |
| 1259-1305 | UGUUUUUGAGAUAGGAGCUUAUUAUGGUACCCCAGGCUGGCUUUGAA | -10.31 |
| 1262-1338 | UUUUGAGAUAGGAGCUUAUUAUGGUACCCCAGGCUGGCUUUGAACUCAAUAUAAUCC | -6.30 |
| 1263-1299 | UUUGAGAUAGGAGCUUAUUAUGGUACCCCAGGCUGGC | -17.60 |
| 1273-1309 | GAGCUUAUUAUGGUACCCCAGGCUGGCUUUGAACUCA | -9.50 |
| 1285-1339 | GUACCCCAGGCUGGCUUUGAACUCAAUAUAAUCCUGCCUUAGCCUUUUCCAAGUU | -5.60 |
| 1285-1307 | GUACCCCAGGCUGGCUUUGAACU | -9.50 |
| 1286-1309 | UACCCCAGGCUGGCUUUGAACUCA | -9.50 |
| 1288-1308 | CCCCAGGCUGGCUUUGAACUC | -9.50 |
| 1290-1346 | CCAGGCUGGCUUUGAACUCAAUAUAAUCCUGCCUUAGCCUUUUCCAAGUUCUGGGGU | -11.80 |
| 1290-1309 | CCAGGCUGGCUUUGAACUCA | -9.50 |
| 1291-1345 | CAGGCUGGCUUUGAACUCAAUAUAAUCCUGCCUUAGCCUUUUCCAAGUUCUGGGG | -9.68 |
| 1292-1323 | AGGCUGGCUUUGAACUCAAUAUAAUCCUGCCU | -10.24 |
| 1296-1350 | UGGCUUUGAACUCAAUAUAAUCCUGCCUUAGCCUUUUCCAAGUUCUGGGGUUACA | -14.05 |
| 1316-1361 | UCCUGCCUUAGCCUUUUCCAAGUUCUGGGGUUACAGGUAUGCACCA | -10.58 |
| 1319-1256 | UGCCUUAGCCUUUUCCAAGUUCUGGGGUUACAGGUAUG | -10.58 |
| 1324-1361 | UAGCCUUUUCCAAGUUCUGGGGUUACAGGUAUGCACCA | -14.05 |
| 1329-1361 | UUUUCCAAGUUCUGGGGUUACAGGUAUGCACCA | -14.05 |
| 1333-1361 | CCAAGUUCUGGGGUUACAGGUAUGCACCA | -14.05 |
| 1340-1363 | CUGGGGUUACAGGUAUGCACCAGC | -13.63 |

Supplementary Table 5: Predicted ncRNA hybridization regions of Bcl-2 mRNA and △G value for circRasGEF1B-Bcl-2 mRNA duplexes

| ncRNA hybridization regions (nt) | Sequences | △G (kcal/mol) |
| --- | --- | --- |
| 454-476 | GUCUCUCUGCCCUGGAGGUCUGA | -27.20 |
| 599-618 | CUGCCGCCGCCGCCGCUGCC | -21.06 |
| 1275-1301 | UCCAAGAAUGCAAAGCACAUCCAAUAA | -7.90 |
| 2449-2470 | CACCUGCUGGCCUGUGCCUGUG | -25.54 |
| 3168-3190 | AGCAGCUGGUCUCUGAGCUCAGA | -24.50 |
| 4207-4228 | CAUUUAACUCAAGUUACCUAGG | -10.01 |
| 4282-4338 | UGGGAAGCAGAGCAAGGGGUUAGGACCUGGCUCUAGCAGCUGGGCCUUCAGAGGGUU | -19.23 |
| 4465-4491 | GGUCUCAGAGAACAGGAUGAUCAGAAC | -11.30 |
| 4471-4807 | UGUAUAUACAUUUAAAAGAAGAAGCAGCAGCAGCUCA | -5.50 |
| 4692-4729 | AUUUUAAAUAUAAAACCUGUUUCUCUUUUGUUUUUGUU | -13.46 |
| 4710-4735 | GUUUCUCUUUUGUUUUUGUUGCUAUU | -21.76 |
| 4715-4736 | UCUUUUGUUUUUGUUGCUAUUG | -15.46 |
| 4717-4738 | UUUUGUUUUUGUUGCUAUUGUU | -13.90 |
| 5744-5774 | CCUACCCUAUCAGGAAAAAAUAACAGGAAAA | -17.80 |
| 5766-5804 | ACAGGAAAAGGUUGAAAUAUAAGCCAGUCUAAGGAAAUU | -13.58 |
| 5820-5842 | UUCUCUUCUGAGCUUAUACUAUG | -13.27 |
| 5862-5891 | CAAAUAUGAUCACACACUUUUUAAGAAAUA | -4.24 |
| 6364-6402 | CAACAUAGAAAAAAUUCAAUAAGAUUGAAUUAAGUAAAA | -10.71 |
| 6718-6749 | UUGGGAACCUGCAGUGGGCCCUCCAGCUGGCU | -9.30 |
| 6773-6795 | GAGAUAACAUCUGAAGGAUUGAU | -14.30 |
| 6821-6848 | UAUUUUCCUUUGGAGACAGGGGUUCUCU | -21.88 |
| 6821-6846 | UAUUUUCCUUUGGAGACAGGGGUUCU | -21.62 |
| 6829-6850 | UUUGGAGACAGGGGUUCUCUAU | -17.48 |
| 6965-6986 | AAGAUUUUCAUACUUAUAUCCA | -6.60 |
