# Supplementary material for: SM22α Loss Contributes to Apoptosis of Vascular Smooth Muscle Cells via Macrophage-Derived circRasGEF1B
Source: Oxid Med Cell Longev. 2021 Mar 16;2021:5564884. doi: 10.1155/2021/5564884 (PMC8026322; doi:10.1155/2021/5564884)
Supplement: Supplementary Materials — Supplementary Table 1: PCR primer sequence. Supplementary Table 2: list of enriched (P < 0.05) differentially expressed proteins related to cellular adhesion identified by SM22α knockout in VSMC conditional media. Supplementary Table 3: list of enriched (P < 0.05) differentially expressed genes related to apoptosis identified by circRasGEF1B knockdown in RAW264.7 cells. Supplementary Table 4: predicted ncRNA hybridization regions of ZFP36 mRNA and ΔG value for circRasGEF1B-ZFP36 mRNA duplexes. Supplementary Table 5: predicted ncRNA hybridization regions of Bcl-2 mRNA and ΔG value for circRasGEF1B-Bcl-2 mRNA duplexes. [file 5564884.f1.zip › Supplementary description.docx]

**Supplementary data**

Supplementary Table 1: PCR primer sequence

Supplementary Table 2: List of enriched (*p* < 0.05) differentially expressed proteins related to cellular adhesion identified by SM22α-knockout in VSMC conditional media.

Supplementary Table 3: List of enriched (*p* < 0.05) differentially expressed genes related to apoptosis identified by circRasGEF1B-knockdown in RAW264.7 cells.

Supplementary Table 4: Predicted ncRNA hybridization regions of ZFP36 mRNA and △G value for circRasGEF1B-ZFP36 mRNA duplexes

Supplementary Table 5: Predicted ncRNA hybridization regions of Bcl-2 mRNA and △G value for circRasGEF1B-Bcl-2 mRNA duplexes
